# Genomic insights into *Ceratobasidium* sp. associated with vascular streak dieback of woody ornamentals in the United States using a metagenomic sequencing approach

**DOI:** 10.1128/spectrum.02523-25

**Published:** 2026-01-15

**Authors:** Kassaye H. Belay, Sahar Abdelrazek, Sehgeet Kaur, Reza Mazloom, Devin Bily, Tashi Gyatso, Farhat A. Avin, John Bonkowski, Prabha Liyanapathiranage, Lina Rodriguez Salamanca, Lenwood S. Heath, Fulya Baysal-Gurel, Boris A. Vinatzer

**Affiliations:** 1 School of Plant and Environmental Sciences, Virginia Tech 1757, Blacksburg, Virginia, USA; 2 Graduate Program in Genetics, Bioinformatics, and Computational Biology, Virginia Tech 1757, Blacksburg, Virginia, USA; 3 Department of Biomedical Sciences and Pathobiology, Virginia-Maryland College of Veterinary Medicine, Virginia Tech 229659, Blacksburg, Virginia, USA; 4 Department of Plant Pathology, Faculty of Agriculture, Assiut University, Assiut, Egypt; 5 Department of Computer Science, Virginia Tech 1757, Blacksburg, Virginia, USA; 6 Virginia Department of Agriculture and Consumer Services 110252, Richmond, Virginia, USA; 7 Otis L. Floyd Nursery Research Center, Tennessee State University, McMinnville, Tennessee, USA; 8 Department of Botany and Plant Pathology, Purdue University 124521 , West Lafayette, Indiana, USA; 9 Ellington Agricultural Center, Tennessee Department of Agriculture 54100, Nashville, Tennessee, USA; Connecticut Agricultural Experiment Station, New Haven, Connecticut, USA

**Keywords:** emerging disease, metagenomic sequencing, woody ornamentals, phylogenetics, pangenome analysis

## Abstract

Woody ornamentals are integral to urban landscapes and play important roles in habitat restoration and ecological conservation, yet their national and international trade facilitates the spread of plant diseases with significant ecological and economic consequences. Vascular streak dieback (VSD) recently emerged on woody ornamentals in the United States and was found to be associated with the fungal pathogen *Ceratobasidium* sp. (*Csp*), but little is known about its genomic diversity and associated microbial communities. We thus applied metagenomic sequencing to 106 symptomatic samples that had tested positive for *Csp* and had been collected from 34 woody ornamental species in seven states. Taxonomic profiling identified *Csp* as the only putative pathogen of which we recovered 17 high-quality draft genomes. Phylogenomic and pangenome analyses revealed that U.S. *Csp* isolates form a tight genetic cluster, distinct in gene content from *C. theobromae*, a pathogen of cacao, avocado, and cassava in Southeast Asia. Comparative analyses highlighted gene content differences, including candidate effectors and secondary metabolite clusters, which may underlie host interactions and offer diagnostic targets. These findings provide the first genomic insights into the U.S. *Csp* population, suggest the recent introduction of a single genetic lineage with a broad host range, and establish a framework for improved detection, monitoring, and management of VSD in woody ornamentals.

**IMPORTANCE:**

Identification of the pathogen that causes an emerging disease, be it of humans, animals, or plants, is a prerequisite to develop effective treatment and/or management practices and to try to control the disease outbreak to prevent further pathogen spread. Vascular streak dieback (VSD) is an emerging disease of ornamental bushes and trees in the United States. Identification of the pathogen has been hindered by the difficulty in growing the fungal pathogen found to be associated with diseased plants in pure culture. Here, we succeeded in sequencing the DNA of the likely pathogen directly from plant tissue or from the fungal mass growing out of collected plant tissue. The sequences were assembled into genomes, which allowed us to precisely identify the pathogen, compare it to related pathogens of other plants, and predict how it causes disease. These results can now be used to inform management and control of VSD.

## INTRODUCTION

Plant pathogenic fungi in the genus *Ceratobasidium* are known to cause severe vascular diseases in Southeast Asia, notably vascular streak dieback (VSD) in cacao (*Theobroma cacao*) and avocado (*Persea americana*) (1, 2) and witches’ broom disease in cassava (*Manihot esculenta*) (3). VSD is characterized by progressive dieback of shoots and branches, often accompanied by leaf yellowing, marginal scorching, and premature defoliation. Infected trees typically show vascular discoloration extending longitudinally in the xylem, which manifests as reddish-brown streaks when stems are split open (1, 4, 5). As the disease advances, plants exhibit stunting, wilt, and canopy thinning, and severely affected trees may experience whole-branch dieback and eventual mortality (4, 5). Recently, VSD has also been reported in woody ornamental species across the United States. A *Ceratobasidium* sp. (*Csp*) was first detected in eastern redbud (*Cercis canadensis* L.) with VSD symptoms in Tennessee in 2019, while similar symptoms were observed in maple (*Acer* spp.) in North Carolina as early as 2018 (6, 7). To date, the disease has been reported in over 12 states and 50 woody plant genera, with the highest incidence in eastern redbud (*Cercis canadensis* L.), followed by maple (*Acer* spp.) and dogwood (*Cornus* spp.) (6, 8, 9). Woody ornamentals play a crucial role in landscaping, habitat restoration, and ecological conservation, and their cultivation and sale significantly contribute to the economic viability of the U.S. nursery industry. The continued spread of VSD is thus a major concern from both an ecological and an economic perspective (10).

*Csp* detection in the United States is currently based on a PCR assay that uses primers specific to the *Csp* ITS region (11). Phylogenetic reconstruction has been performed using the ITS, LSU, ATP6, TEF1, and RPB2 genes and revealed a close genetic relationship between *Csp* in the United States and *C. theobromae* (*Ct*) from cacao and cassava in Southeast Asia, but since *Csp* sequences form a distinct clade, they may represent a separate, previously unrecognized species (6). Also, the 100% sequence identity in the ITS region between the U.S. *Csp* sequences and *Ct* sequences from Japanese honeysuckle (*Lonicera japonica*) in China suggests that *Csp* in the United States may be more closely related to *Ct* in China than to *Ct* in Southeast Asia (6). Koch’s postulates have not been completed because of the fastidious nature of *Csp*. Mycelium grows out of plant tissue but, so far, is recalcitrant to sub-culturing. Therefore, it cannot be excluded that other biotic or abiotic factors contribute to the disease.

The genera *Ceratobasidium* and *Rhizoctonia* are closely related basidiomycete fungi within the family *Ceratobasidiaceae*, exhibiting overlapping morphological and ecological traits that complicate their differentiation (12). Historically, many *Csp* were identified as the sexual (teleomorphic) stage of *Rhizoctonia* (13, 14). Recent phylogenetic investigations have further blurred genus boundaries, revealing that some species formerly classified under *Ceratobasidium* are more accurately placed within *Rhizoctonia* (12, 13). Regarding pathogenicity, *Rhizoctonia* is notorious for causing a broad range of diseases such as damping-off and root rot (12). In contrast, *Csp* generally adopt a more specialized biotrophic lifestyle, maintaining a more intimate and often less aggressive interaction with their hosts (1, 2).

Genomic studies of *Ct* have greatly advanced understanding of this pathogen’s biology and evolution (15, 16). These studies revealed compact genome sizes of approximately 33–34 Mb encoding 9,000–10,000 genes. Comparative genomic analyses identified around 800 genes unique to *Ct* compared to its closest relatives, potentially underpinning *Ct*’s vascular pathogenicity, including an extensive secretome, plant cell-wall-degrading enzymes, and virulence-associated factors such as toxin biosynthesis and effector proteins (15, 16).

A comprehensive understanding of the emergence, distribution, transmission, and evolutionary dynamics of emerging plant diseases, such as VSD, is essential for developing effective disease containment or management strategies (17, 18). Today, for culturable pathogens, this information can be obtained by whole-genome sequencing (WGS) with long-read platforms, like PacBio and Oxford Nanopore Technologies, which facilitate accurate assembly and characterization of complex fungal genomes (19, 20). However, WGS becomes challenging or impossible to use in the case of fastidious or non-culturable pathogens, like *Csp*. Metagenomic sequencing, that is, the direct sequencing and analysis of all genetic material obtained from an environmental or host sample without requiring cultivation (21), can potentially provide the same information as WGS. It can even identify mixed infections. But metagenomics is faced with several challenges. In the case of plant samples, an overabundance of host sequences limits the number of pathogen sequences that can be obtained. Also, the presence of many other microbes may make it challenging to separate pathogen sequences from non-pathogen sequences, which limits the possibility of obtaining a complete pathogen genome with minimal contamination. Therefore, metagenome-assembled genomes (MAGs) of plant pathogens have so far mainly been obtained for bacteria (22–26).

To start answering the open questions about VSD on woody ornamentals in the United States, here, we sequenced 106 samples from plants with VSD symptoms that had been confirmed to be infected with *Csp* with a PCR assay (6, 11), including 61 symptomatic vascular tissue samples and 45 mycelia from 34 different woody plant species collected in seven U.S. states. The main objectives were to (i) perform a survey of bacterial, fungal, and oomycete pathogens present in symptomatic vascular tissues to identify the microbial diversity in VSD symptomatic tissue, including the detection of *Csp* and other co-existing microorganisms, (ii) assemble *Csp* genomes from plant metagenomic and mycelium samples to elucidate evolutionary relationships among *Csp* sequences from the United States and with *Ct* sequences from Southeast Asia, and (iii) assess differences in gene content, including predicted virulence genes, between *Csp* and *Ct* to start unraveling potential adaptation of *Csp* to woody ornamentals and the various regional climates in North America.

## MATERIALS AND METHODS

### Plant sample collection

Survey sites included wholesale and retail nurseries, along with landscape sites with newly planted trees. Symptomatic native plants located near landscape trees or nursery stock at each survey site were also sampled. Sampling was performed as described previously (8). In short, one to three plant samples were chosen from a block, group, or cultivar of plants that best represented the symptoms observed, which included marginal leaf scorching, leaf chlorosis, stunting, wilt, vascular discoloration, and dieback. These symptoms were consistently observed across all samples at each site. Symptomatic plant parts or the entire plant with roots were placed in sealed plastic bags at room temperature and were either shipped overnight or delivered the same day to the laboratory. Fungal cankering and oomycete root rot pathogens were occasionally associated with symptomatic plants. However, note that only plants that tested positive for *Csp* were used further (see next section). Table S1 provides comprehensive information, including host plant species, geographic collection locations, and isolation dates.

### Plant sample processing, culturing, DNA extraction, and PCR

Samples were processed as previously described (8). In short, after removing foliage, stems were cut into 15–20 cm pieces, surface sterilized in 70% ethanol, and incubated at ≤90% humidity at 22°C ± 2°C in the dark for 24–96 h. Using a scalpel, 0.1 g of discolored xylem tissue was cut out lengthwise or sliced into discs from smaller, debarked shoots. If parts of the plant were dead, any discolored living tissue at the margin between dead or wilted parts was selected. If the sample lacked dead tissue or vascular discoloration, four pieces (0.025 g each) of xylem tissue would be selected every 5 cm and combined, making sure to select from multiple growth rings. Tissue was then macerated in CD1 buffer of the Qiagen DNeasy Plant Pro Kit (Germantown, MD) with a BioSpec MiniBeadbeater-16 (Bartlesville, OK) or MP Biomedica FastPrep-24 5G (Solon, OH), followed by DNA extraction using the manufacturer’s protocol. Conventional PCR and qPCR targeting *Csp* were used on extracted DNA as previously described (8, 11).

If stems had sufficient moisture content, culturing from vascular tissue was attempted, where a 4–5 mm piece of discolored xylem tissue was surface sterilized with 2.5% NaOCl, again with 70% ethanol, and air-dried before being placed on water agar medium (pH 7.1) and incubated in the dark at 22°C ± 2°C for as long as 15 d, where 0.1 g of *Rhizoctonia*-like mycelia was swabbed with a sterile needle into an extraction tube with CD1 buffer. For other stem samples, mycelium was harvested under a dissection microscope with a sterile needle directly from cut stems, incubated for 96 h. DNA was then extracted from mycelium using the Qiagen DNeasy Plant Pro Kit.

### Computational resources

All bioinformatic analyses were performed on the OWL’s Nest and TinkerCliffs high-performance computing clusters (Advanced Research Computing, ARC) at Virginia Tech. Analyses typically used 20–40 CPUs and up to 256 GB of RAM per job, and all major software tools and pipelines were installed and managed through centrally maintained conda environments.

### Metagenomic sequencing

Long-read sequencing was performed on an Oxford Nanopore Technologies (ONT) PromethION sequencer. Sequencing libraries were prepared following the manufacturer’s protocol for SQK-NBD114.24, provided by ONT. Briefly, nucleic acid extracted from each sample went through an FFPE repair step and then purified using AMPure XP beads (Beckman Coulter Life Sciences, Indianapolis, IN). Following purification, sequencing adapters were ligated to each sample, then subjected to a final purification step using AMPure XP beads. For each sequencing library that was prepared, barcoded and adapter-ligated samples were pooled together and eluted in 25 µL of ONT elution buffer. PromethION flow cells (FLO-PRO114M) were primed, and the sequencing library concentration and quality were assessed using Qubit and TapeStation, then adjusted to meet PromethION requirements before being loaded into the primed flow cells for sequencing. The resulting FASTQ files from each flow cell were concatenated into a single file per sample for downstream analysis. All reads that passed ONT quality control (fastq_pass) were utilized in subsequent analyses. ONT reads were basecalled using Guppy v6.3.7 in high-accuracy mode, and reads were quality filtered with NanoFilt v2.8.0 using a minimum average Phred quality score of Q ≥ 10 and a minimum read length of 1,000 bp. Reads not meeting these thresholds were discarded. After concatenation, each FASTQ file was evaluated to determine read statistics, including the total number of reads, total base count (the sum of all read lengths), and the average read length (Table S1). These metrics were calculated using a custom Bash script that parsed the FASTQ files by counting sequence lines and summing their lengths to generate the summary statistics.

### Taxonomic profiling

To identify plant host sequences, the read mapping tool Minimap2 (27) was used, choosing as reference genomes the plant genome assembly available in NCBI that was most closely related to the host of isolation (accession numbers are listed in Table S1). Minimap2 was also used to identify *Csp* sequences by mapping reads against the two available *Ct* genomes from cacao: CT2 (accession GCA_009078325.1) and Gudang 4 (accession GCA_012932095.1). Reads that mapped against both genomes were de-duplicated before downstream analysis.

The taxonomic profiler Kraken2 (28, 29, 30) was employed to assign sequencing reads to bacterial, fungal, and oomycete taxa. Two databases were used for identification of bacterial and fungal taxa, respectively: (1) the standard k2_pluspfp_20240605 database, comprising RefSeq archaea, bacteria, viral, plasmid, human, protozoa, fungi, plants, and UniVec_Core (31), and (ii) a custom fungi-oomycete database to address the limited representation of these taxa in RefSeq. To generate the custom database, 18,644 fungal and 513 oomycete genome assemblies were downloaded from NCBI on 24 July 2024. These assemblies were processed using LINflow (32) to assign Life Identification Numbers (LINs). Subsequently, representative genomes were selected at an average nucleotide identity (ANI) threshold of 99.5%, resulting in a non-redundant, comprehensive Kraken2 database containing 8,797 fungal and 342 oomycete genomes.

Additionally, taxonomic profiling was performed using sourmash v4.5.0 (33, 34). Raw sequencing reads were converted into FracMinHash sketches with a k-mer size of 31 and a scaled parameter of 1,000. Bacterial community composition was determined using the sourmash gather command against the GTDB-RS207 reference database. To analyze fungal communities, the genomes used above for the fungi-oomycete Kraken2 database were used to build a custom sourmash database with the same FracMinHash parameters as for bacteria.

### *Csp* genome assembly and analysis

Samples containing more than 10,000 reads mapping to the two *Ct* reference genomes (GCA_009078325.1 and GCA_012932095.1) were selected for genome assembly. Each selected sample was assembled independently using Flye (35), specifying a genome size parameter of 34 Mb based on the *Ct* reference genome size. Five rounds of iterative polishing were conducted to enhance assembly accuracy.

To capture *Csp*-specific genomic regions potentially missed by aligning exclusively to *Ct* genomes, we constructed a comprehensive assembly by pooling the five metagenomic samples containing the highest abundance of *Ct*-specific reads. Host-derived sequences were first removed using Minimap2. The remaining non-host reads were combined and subjected to *de novo* assembly using Flye (35). Following this assembly, initial contig filtering was conducted using Kraken2 with both standard and custom fungi-oomycete databases. Contigs were further analyzed using BLASTn searches (36) against the NCBI core nucleotide database with relaxed alignment parameters, maximizing detection of divergent sequences. Additionally, read-depth consistency analyses were performed on each contig using Samtools (37), CoverM (38), and mosdepth (39). Only contigs displaying high sequence similarity (≥95% nucleotide identity over ≥1 kb) to non-*Ceratobasidiaceae* taxa, or those exhibiting inconsistent read-depth patterns, were excluded. This approach minimized the risk of inadvertently removing genuine Csp sequences.

### Phylogenetic analysis

For core-genome construction, genome completeness was first assessed using BUSCO v5.2.2 with the Agaricomycetes_odb10 data set, comprising 2,898 single-copy orthologs (40). Conserved single-copy orthologs present in all genomes used for tree construction were aligned using MUSCLE (41), and alignments were trimmed with TrimAl (42) to remove poorly aligned regions that could introduce artifacts in phylogenetic reconstruction. A maximum likelihood tree was then inferred using IQ-TREE (43). Branch support was evaluated using ultrafast bootstrap approximations implemented in IQ-TREE (v2.2.0.3) (43) under the VT + F + I + I + R6 model with 1,000 ultrafast bootstrap replicates. The final phylogenetic trees were visualized and annotated in R version 4.2.3 using the ggtree package (44).

For the single-nucleotide polymorphism (SNP) analysis, raw reads were first host-filtered to remove plant sequences. The remaining non-host reads were aligned to two Ct reference genomes, CT2 (GenBank: GCA_014217725.1) and Gudang 4 (GenBank: GCA_012932095.1), to extract *Ceratobasidium* (*Csp*) reads. The extracted *Csp* reads were then mapped to the *Ct* reference isolate LAO1 (GenBank: GCA_037974915) using minimap2 v2.29 with the -ax map-ont preset. Alignments were sorted and indexed with samtools (v1.19.1). Variants were called per isolate using NanoCaller (v3.6.2) in haploid mode (--mode all, --sequencing ul_ont,--snp_model ONT-HG002, --indel_model ONT-HG002), producing one VCF containing SNPs and indels per sample. VCFs were coordinated‐sorted, BGZF‐compressed, and indexed with bcftools and tabix, then merged into a single multi‐sample VCF (bcftools merge --force-samples). The merged VCF was filtered to retain only high‐confidence SNPs (per‐site QUAL ≥20 and per‐sample depth DP ≥ 10) using bcftools view -i “QUAL>=20 && FMT/DP>=10.” The resulting filtered VCF was converted to a multi‐FASTA alignment in PHYLIP format using a custom vcf2phylip script. A maximum‐likelihood phylogeny was inferred in IQ-TREE (v2.2.0.3) (43) under the GTR+Γ model with 1,000 ultrafast bootstrap replicates. The final phylogenetic tree was visualized and annotated in R version 4.2.3 using the ggtree package (44).

### Gene prediction and comparative genomics

Gene prediction was performed using AUGUSTUS v3.5.0 (45) with an *ab initio* approach trained on the *Rhizoctonia solani AG-1 IA* genome (GenBank: GCF_016906535.1). We trained the classifier on *R. solani* due to its well-curated genome and comprehensive effector predictions, whereas *C. theobromae* currently lacks equivalent functional annotation, limiting its utility for reliable training and benchmarking. The training pipeline began by initializing species-specific configuration files using new_species.pl to establish baseline parameters, followed by automated gene model training with autoAugTrain.pl utilizing multicore optimization. Pre-processing of annotations retained only mRNA and CDS features to ensure AUGUSTUS training compatibility, resulting in optimized gene model parameters for accurate prediction in *Csp* genomes. Following gene prediction, protein-coding sequences were extracted from GFF3 annotations using gffread (46) and underwent quality control curation. OrthoFinder (47) performed orthologous clustering using the DIAMOND (48) algorithm in a multi-threaded configuration, while custom Python scripts generated presence/absence matrices to define core and accessory orthogroups with gene IDs mapped to their respective genomes. Core orthogroup protein sequences were concatenated. The concatenated core protein sequences underwent MAFFT (49) alignment using 40 threads for optimal performance, followed by TrimAl processing to remove poorly aligned regions that could interfere with phylogenetic signals. Seqmagick converted the trimmed alignment into PHYLIP format for compatibility with downstream analysis tools. IQ-TREE performed maximum likelihood phylogenetic reconstruction using the VT + F + I + I + R6 substitution model, with branch support assessed through both ultrafast bootstrap and SH-aLRT (1,000 replicates each), ultimately providing evolutionary relationship insights among *Csp* isolates.

### Functional genome annotation

Protein sequences from core and accessory genomes were functionally annotated using InterProScan (50). Before annotation, sequences were quality-checked to retain only valid amino acid residues. InterProScan analyses utilized multiple protein databases (Pfam, SMART, CDD, etc.) to annotate protein domains, GO terms, and metabolic pathways. InterProScan was executed on high-performance computing resources, allocating 150 GB RAM and 16 CPU cores. Outputs included TSV, XML, and GFF3 formats, providing extensive functional annotations suitable for downstream enrichment and comparative analyses. Quick summary tables were generated to count the frequency of InterPro domains, GO terms, and associated pathways, facilitating functional enrichment analyses to identify potential host-specific adaptations and evolutionary innovations in *Csp* genomes.

### Prediction and characterization of putative effector proteins

Putative effector proteins were identified from the predicted accessory proteome following a multi-step computational pipeline integrating secretion, localization, and functional prediction tools (51, 52). Candidate protein sequences derived from accessory orthogroups were first screened for the presence of N-terminal signal peptides using SignalP v6.0 (53) in eukaryotic mode with fast processing settings. Signal peptide-positive sequences were then analyzed with TMHMM v2.0c (54, 55) in short output mode to identify proteins containing transmembrane helices. Proteins predicted with one or more helices were excluded to retain only putative soluble secreted proteins. Proteins lacking transmembrane helices were further screened using TargetP v2.0 (56) in non-plant eukaryotic mode to identify and exclude proteins predicted to contain mitochondrial targeting peptides (mTP). The remaining secretome candidates were scanned for endoplasmic reticulum retention signals (PS00014 motif) using ScanProsite (57). Proteins carrying this motif were excluded to avoid ER-retained proteins. The refined secretome was finally evaluated for glycosylphosphatidylinositol (GPI) anchor signals using NetGPI v1.1 (58). Candidate proteins were split into batches and submitted to the web server for prediction. Proteins identified as GPI-anchored were excluded from the secretome. The filtered secretome was submitted to EffectorP v3.0 (59) in fungal mode to predict candidate effector proteins. EffectorP provided classification outputs distinguishing effector and non-effector proteins, along with localization categories as apoplastic, cytoplasmic, or dual-localized effectors. EffectorP prediction outputs were parsed to separate candidate effectors by localization class. Protein sequences corresponding to each effector class were extracted using seqkit (60) and compiled into dedicated FASTA files.

## RESULTS

### Collection of 106 symptomatic *Csp*-positive samples from 34 plant species in seven U.S. states

**Fig 1:**
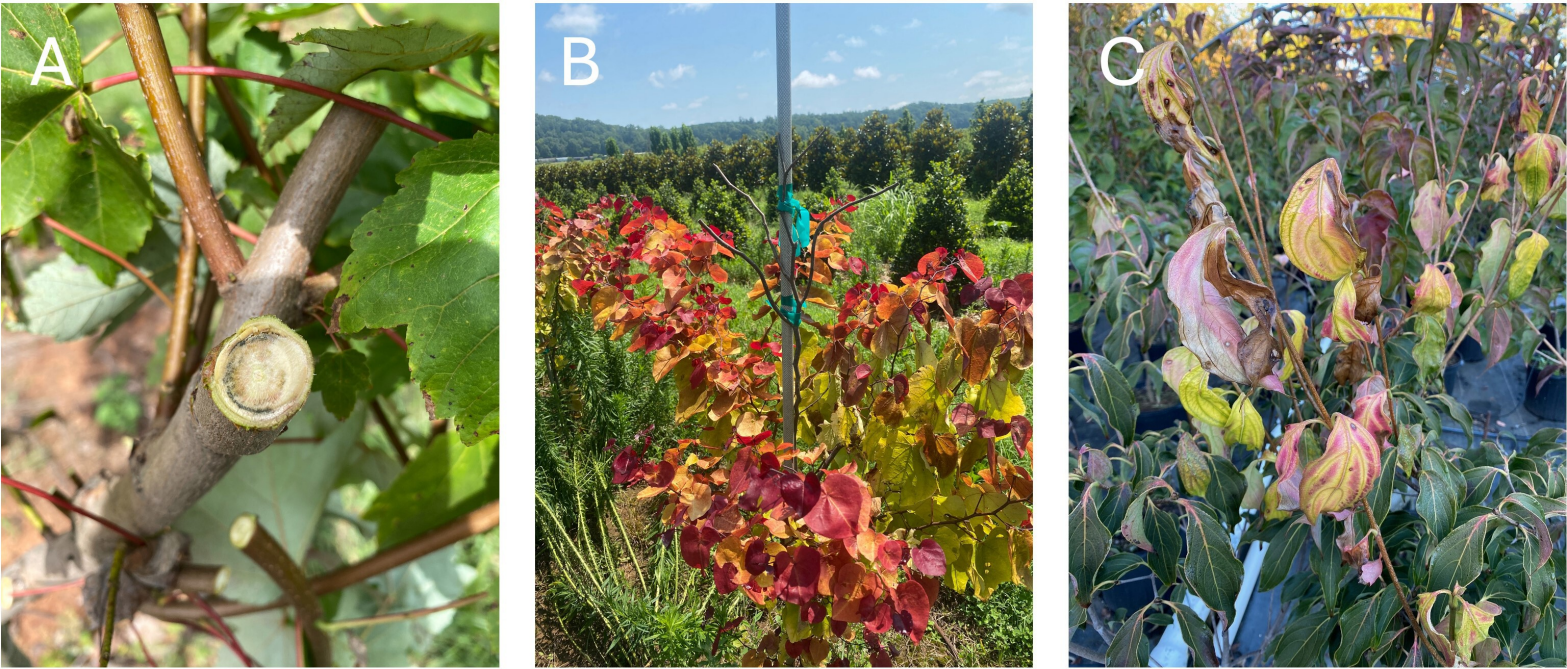
Representative VSD symptoms observed in *Csp*-positive woody ornamental hosts: (**A**) *Acer rubrum* “October Glory”; (**B**) *Cercis canadensis* “Flame Thrower”; (**C**) *Cornus kousa* “Greensleeves.” Symptoms include vascular discoloration, foliar chlorosis progressing to necrosis, and terminal dieback.

**Fig 2:**
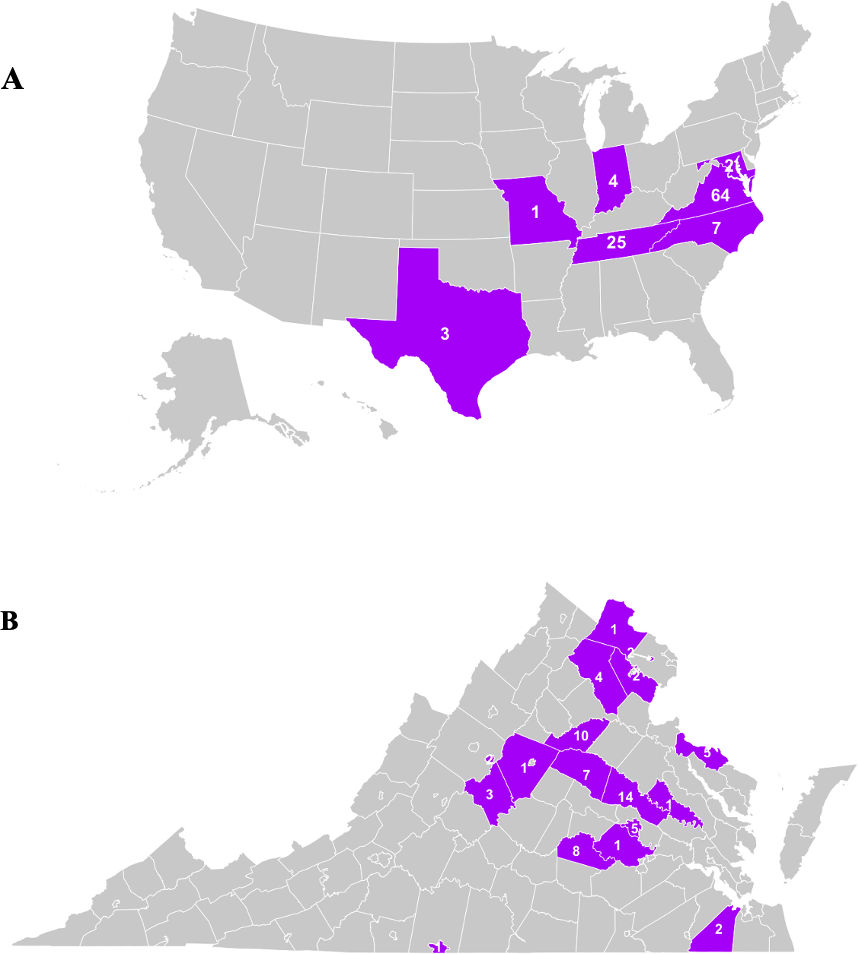
Geographic distribution of *Csp* sample collection sites across the United States. (**A**) Map of the United States with the states from which samples were obtained highlighted in purple. The numbers indicate the number of samples collected from each state. (**B**) A map of Virginia showing counties and the number of samples that were collected.

Plant tissue specimens exhibiting VSD symptoms were collected from various woody ornamental hosts across seven U.S. states. Sampling criteria included the presence of vascular discoloration, foliar chlorosis progressing to necrosis, and terminal dieback consistent with previously documented VSD symptoms (Fig. 1). The frequency of symptoms varied with nursery, ranging from a few scattered plants to 90% of the stock. A total of 106 samples were obtained and tested positive for *Csp* based on a diagnostic PCR assay (11). Virginia contributed the largest sample cohort (*n* = 64, 58.2%), followed by Tennessee (*n* = 25, 22.7%), North Carolina (*n* = 7, 6.4%), Indiana (*n* = 4, 3.6%), Texas (*n* = 3, 2.7%), Maryland (*n* = 2, 1.8%), and Missouri (*n* = 1, 0.9%) (Fig. 2A). Within Virginia, specimens were collected from 14 counties with the greatest concentration in the central Piedmont region (Orange, Louisa, Fauquier, Nelson, Loudoun, and Albemarle) (Fig. 2B), which contains the highest density of commercial woody ornamental nurseries in the state.

The samples originated from 34 woody ornamental host species belonging to 19 different plant families (Table S1), with eastern redbud (*Cercis canadensis*, *n* = 28), Freeman maple (*Acer × freemanii*, *n* = 18), and flowering dogwood (*Cornus florida*, *n* = 16) comprising the majority of specimens. Additional hosts included, but were not limited to, Japanese maple (*Acer palmatum*), southern wax myrtle (*Morella cerifera*), Chinese dogwood (*Cornus kousa*), crepe myrtle (*Lagerstroemia indica*), and various oak species (*Quercus* spp.).

### Deep metagenomic sequencing provides insight into microbial communities associated with VSD

Sequencing was performed on DNA either extracted from vascular tissue (61 samples) or from mycelium cultured directly from vascular tissue (45 samples). Sequencing yielded a cumulative total of 188,856,320 reads across all samples. The sequencing depth per sample was highly variable, ranging from 14,364 to 7,555,390 reads, with an average of 1,760,656 reads (see detailed sequencing results for each sample in Table S1). The mean read length for all sequencing runs was 2,940 base pairs (bp), with the longest obtained read being 396,120 bp long, resulting in a combined total of approximately 893 gigabases (Gb) of sequence data. The high sequencing depth in many samples promised to enable comprehensive profiling of microbial communities and robust detection of *Csp* even in samples with low *Csp* abundance.

**Fig 3:**
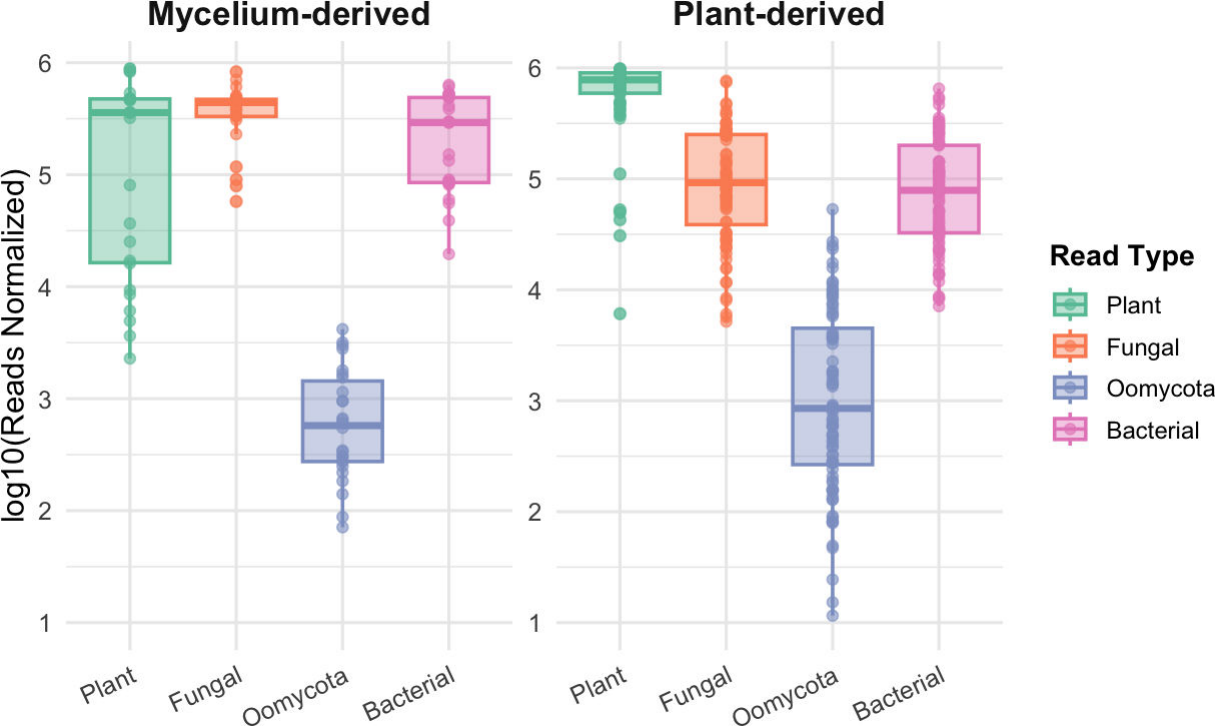
Boxplots of log₁₀-transformed normalized read counts (reads per million) for plant, fungal, Oomycota, and bacterial taxa, comparing mycelium-derived (left) and plant-derived (right) samples.

Not surprisingly, plant reads represented the majority of reads in sequenced plant samples (67.8% on average, with a range from 50.01% to 99.01%) but plant reads also accounted for a substantial fraction of mycelium-derived samples averaging 31.7%, with a range 0.3%–88.2% (Fig. 3). This was likely because mycelium frequently grew intermixed with remnants of vascular plant tissue and small amounts of host material were occasionally co-harvested during sample preparation. As expected, fungal and bacterial read counts were higher in mycelium compared to plant samples (approximately 10-fold) because of the lower average number of plant reads. However, in both sample types, bacterial read and fungal read numbers were similar to each other. Reads assigned to oomycetes were overall much lower, but relatively higher in plant samples compared to mycelium samples.

**Fig 4:**
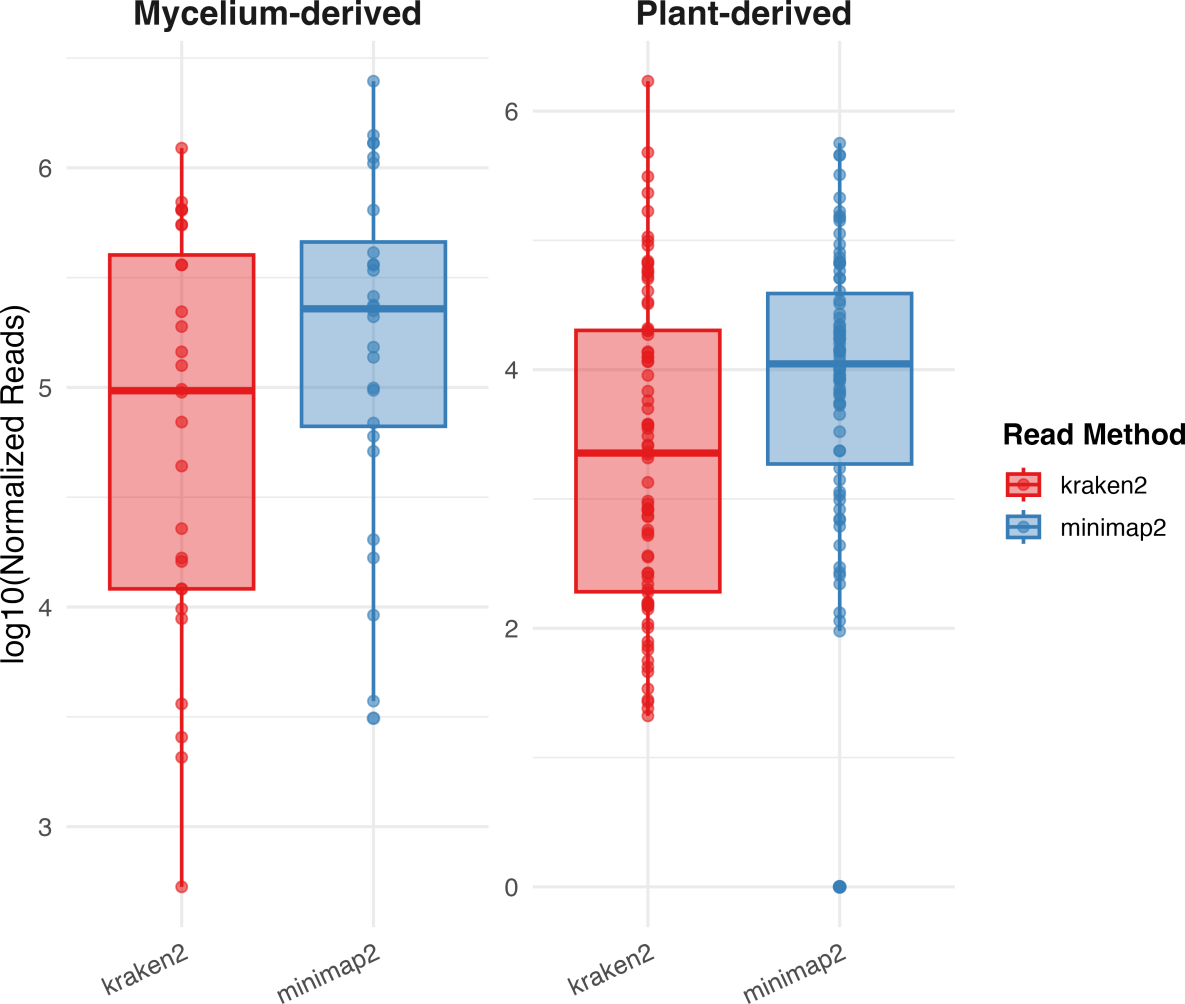
Boxplots of log₁₀-transformed normalized read counts of *Csp* comparing Kraken2 (red) and Minimap2 (blue) across mycelium-derived (left) and plant-derived (right) samples. In both sample types, Minimap2 shows a slightly higher median and tighter distribution of read counts than Kraken2.

**Fig 5:**
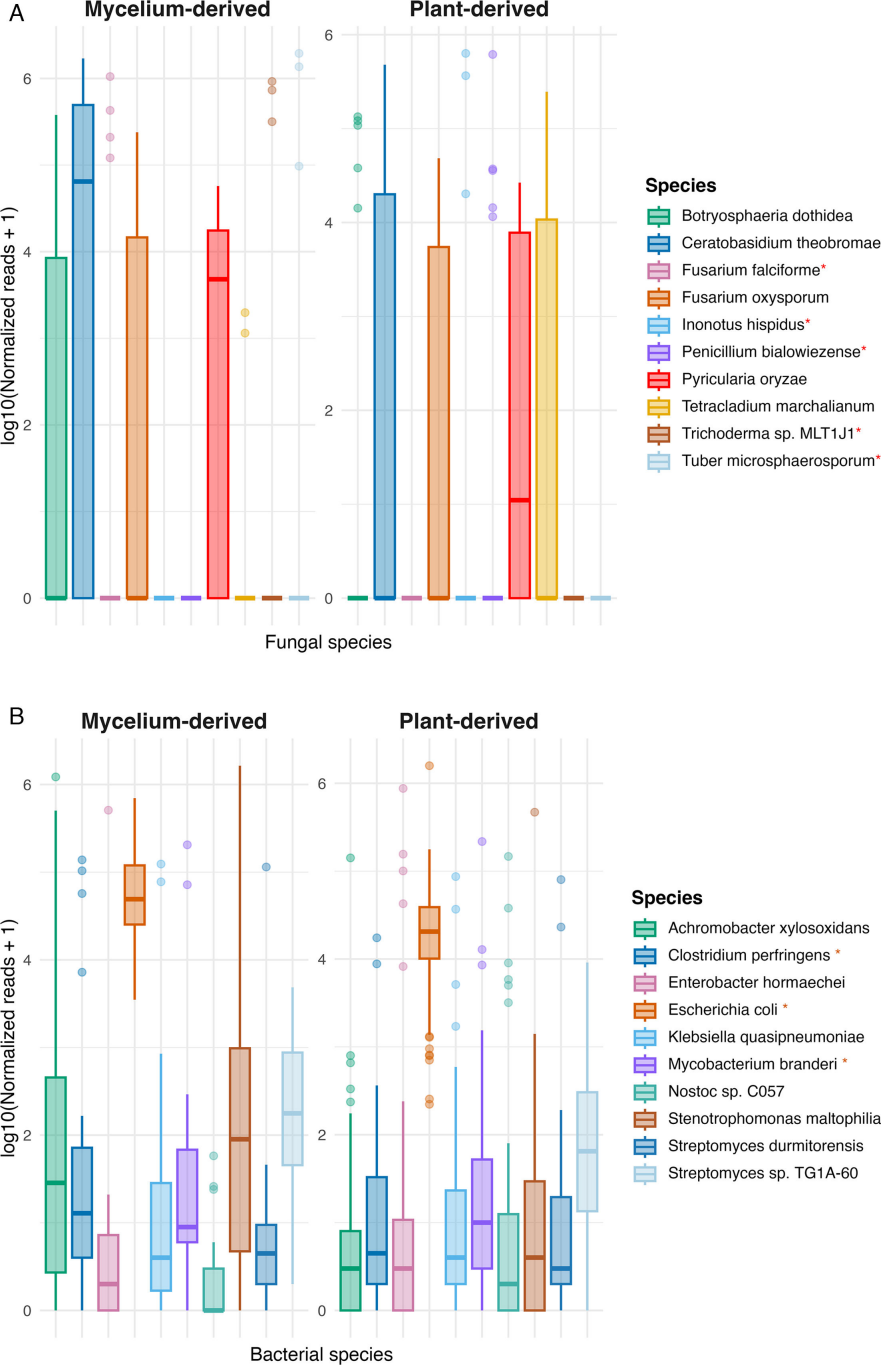
Two-panel boxplots of log₁₀-transformed normalized reads for the top 10 taxa identified by Kraken2. (**A**) Fungal species and (**B**) bacterial species, each comparing mycelium-derived (left) and plant-derived (right) samples. Boxes show the interquartile range with the median (thick line); whiskers extend to 1.5 × IQR; points are per-sample values. Species marked with an asterisk (*) in the legends were not supported by read-mapping or sourmash and are therefore likely Kraken2 false positives.

Previous investigations of VSD in woody ornamentals in the United States focused on the fungal pathogen *Csp* (6, 8, 11). Here, metagenomic sequencing allowed us to investigate the entire microbial communities associated with VSD and determine whether any other microbe consistently co-occurs with *Csp*. Fungal taxonomic classification using the Kraken2 tool (28–30) with a custom fungal and oomycete database revealed distinct fungal community patterns between mycelium and plant samples. In mycelium samples, *Csp* was the dominant taxon, ranking first in 23 of the 30 samples with relative abundance from 19.6% to 91.9%, exceeding half of all non-host reads in 20 samples. The number of *Csp* reads was independently confirmed by mapping reads against *Ct* genomes, which largely aligned with the taxonomic profiling results (Fig. 4; Table S1). Based on Kraken2, *Fusarium falciforme*, a pathogen that causes wilt disease and root rots in many herbaceous and some woody species (61, 62), was the dominant taxon in the remaining mycelium samples (Fig. 5A; Table S1). However, when mapping these reads against a *F. falciforme* reference genome, on average, only 0.3% of reads identified by Kraken2 as *F. falciforme* mapped to *F. falciforme* genomes. When these reads were assembled into contigs, the contigs could not be confirmed by BLAST searches against a custom *Fusarium* database. Further validation using the taxonomic profiler sourmash (33, 34) with a custom fungi and oomycete database containing the same genomes used for the Kraken2 database also failed to detect *F. falciforme* (Table S2).

Plant samples exhibited greater variability regarding the most highly abundant fungal species than mycelium samples (Fig. 5A; Table S1). Only in 16 of 76 plant samples was *Csp* the most abundant fungus, with relative abundances ranging from 6.33% to 82.46% out of all non-host reads. As in mycelium samples, when *Csp* was not dominant, *F. falciforme* was often the most dominant species based on Kraken2 (but, as for mycelium samples, these reads could not be confirmed). *Botryosphaeria dothidea*, a canker and dieback pathogen of trees with a broad host range (63) and previously excluded from causing VSD (6), was found to be abundant in 13 plant samples. Although *B. dothidea* was confirmed by both sourmash and read mapping against a *B. dothidea* reference genome, when attempting assembly of *B. dothidea* reads, only highly fragmented short assemblies were obtained, suggesting lower abundance than that inferred by Kraken2 (Table S2). *Pyricularia oryzae* was another species frequently found among the most abundant taxa, ranking within the top three in 58 plant samples. The presence of *P. oryzae* was confirmed using the taxonomic profiler sourmash, but reads extracted by mapping to a reference genome could not be assembled into any contigs, indicating an overestimation of abundance and likely misclassification by Kraken2 and sourmash (Table S2). Other fungal species detected by Kraken2 included the endophyte *Tetracladium marchalianum*, the broad host-range pathogen *Fusarium oxysporum*, the environmental fungus *Penicillium bialowiezense*, the powdery mildew pathogen *Erysiphe pulchra*, the mushroom *Psilocybe semilanceata*, and the endophyte (and occasionally pathogen) *Trichoderma* spp. (Fig. 5A; Table S1). However, none of the pathogens in this list were present with enough consistency and sufficient abundance to suggest them as contributing to VSD. Moreover, follow-up analyses did not confirm their presence at the levels of abundance that Kraken2 determined (Table S2).

Oomycetes represented a minor component of the non-host reads in both sample types based on Kraken2 using our custom fungi-oomycetes database, accounting on average for only 1.08% of all non-host reads in plant samples and for only 0.36% in mycelium samples. *Plasmopara halstedii* and *Plasmopara obducens* were the most frequently detected species across both sample types (Table S1). Other regularly detected oomycetes included *Pseudoperonospora cubensis* and various *Phytophthora* species. We thus conclude that oomycetes are unlikely to play a role in VSD.

Metagenomic analysis of bacteria revealed 37 genera among the top three most abundant bacterial taxa across samples (Table S1). Relative abundances varied between plant samples and mycelium samples. In plant samples, members of the *Enterobacteriaceae,* including *Enterobacter hormaechei*, *Escherichia coli*, and *Klebsiella quasipneumoniae,* were among the most prevalent, while mycelium-derived samples were characterized by higher relative abundances of *Stenotrophomonas maltophilia, Clostridium perfringens*, and *Achromobacter xylosoxidans* (Fig. 5B; Table S1). To validate these taxonomic assignments, we performed read mapping against reference genomes for the five most abundant bacterial species. Of these, only assignments to *Achromobacter xylosoxidans* and *Stenotrophomonas maltophilia* were confirmed, and enough reads were found to assemble the respective genomes, whose identities were confirmed using genomeRxiv (64). For *Escherichia coli*, results varied significantly across samples, prompting additional verification using the FracMinHash-based sourmash tool, which indicated that most reads assigned to *E. coli* by Kraken2 more closely matched genomes of various environmental and commensal taxa, such as *Methylobacterium* sp., *Bradyrhizobium* sp., and *Curtobacterium* sp., suggesting potential misclassification by Kraken2. Likewise, reads assigned by Kraken2 to *Clostridium perfringens* and *Mycobacterium branderi* aligned to other taxa in the sourmash analysis (Table S3). In summary, no bacterial pathogen was consistently identified across samples, strongly suggesting that bacterial pathogens do not contribute to VSD.

### Genome assemblies of *Csp* isolates

*Csp* reads identified by mapping against the two *Ct* reference genomes were used to construct MAGs. Mapped reads were extracted, deduplicated, and assembled. High-quality draft genome assemblies were obtained from 17 samples (three from plant samples and 14 from mycelium samples), comparable in size and completeness to the *Ct* reference genomes based on BUSCO completeness scores (Table 1). Comprehensive statistics, including read counts, genome completeness, genome sizes, and BUSCO scores, are detailed in Table S4. Twenty-three other samples yielded partial genome assemblies due to lower read counts (fewer than 59,918 reads) with genome sizes ranging from 2.3 to 30.2, while 46 samples had such a small number of *Csp* reads (fewer than 14,177) that no meaningful assemblies were obtained.

**TABLE 1:** Summary statistics of *Csp* genome assemblies and *Ct* reference genomes

Sample ID	Assembly length (Mb)	N50 (bp)	No. of contigs	Annotated genes	No. of singletons	BUSCO (Agaricomycetes_odb10)	BUSCO (Fungi_odb10)	Sample type
23_02068_EasternRedbud_IN	32.97	21,383	2,325	10,442	356	72.7	83.3	PD^*a*^
FBG5639_EasternRedbud_TN	36.94	53,266	2,040	11,655	567	82.0	91.9	PD
RT23_468_FloweringDogwood_VA	38.11	28,656	2,614	10,971	320	78.1	89.3	PD
RT23_372_FloweringDogwood_VA	36.84	161,947	786	10,990	35	83.6	94.2	MD^*b*^
RT23_384_FloweringDogwood_VA	36.78	460,389	562	10,936	8	84.0	94.6	MD
RT23_392_SouthernCatalpa_VA	36.86	549,311	483	10,779	2	83.7	94.5	MD
RT23_402_RedOsierDogwood_VA	36.01	643,614	475	10,887	0	83.9	94.6	MD
RT23_423_ChineseDogwood_VA	36.65	390,318	543	10,678	9	83.9	94.6	MD
RT23_424_ChineseDogwood_VA	35.37	139,030	915	10,919	38	83.6	94.2	MD
RT23_428_ChineseDogwood_VA	37.00	616,318	539	10,939	1	83.8	94.5	MD
RT23_429_ChineseDogwood_VA	36.86	700,228	485	10,880	3	83.9	94.5	MD
RT23_433_FloweringDogwood_VA	36.53	816,925	401	11,141	5	83.9	94.6	MD
VSD24_268_Sourwood_VA	32.89	37,465	1,409	10,217	311	72.2	82.7	MD
VSD24_269_RedOsierDogwood_VA	36.25	266,961	559	10,863	24	83.6	94.6	MD
VSD24_270_FloweringDogwood_VA	36.11	97,071	669	10,913	177	80.4	91.7	MD
VSD24_DSB_110M_SouthernWaxMyrtle_VA	36.82	379,450	293	10,779	28	83.6	94.6	MD
VSVD24_368_EasternRedbud_VA	36.18	320,509	504	10,792	13	83.9	94.6	MD
Comprehensive_assembly	37.75	824,000	518	10,962	5	83.7	94.3	Mixed
*Ct* CT2	31.20	86,100	1,240	9,763	104	83.6	94.5	NCBI
*Ct* Gudang 4	38.60	39,200	4,661	13,311	1,201	82.0	92.2	NCBI

^*a*^ MD, Mycelium-derived.

^*b*^ PD, Plant-derived.

Since construction of the *Csp* MAGs relied exclusively on reads mapped to the *Ct* reference genomes, we were concerned that we may have failed to capture genomic regions potentially present in *Csp* but absent from *Ct*. Therefore, we conducted a second, independent assembly approach to include these potential regions unique to *Csp*. To do this, we selected the five samples with the highest *Ct*-aligned read counts. Instead of assembling mapped reads, we removed host sequences and then assembled all other reads (2,520,850 reads with a total length of 12.1 Gb). The assembly resulted in 522 contigs. We then taxonomically classified these contigs using BLASTn and Kraken2 and removed four contigs that had best hits to non-fungal organisms (Table S5). While detailed assembly results are shown in Fig. S1, Table 1 shows that the comprehensive assembly is very similar in quality to the best individual assemblies. Importantly, the number of annotated genes in the comprehensive assembly (10,962) fell within the range of annotated genes in the individual assemblies (10,442–11,141) and, based on our pangenomes analysis (see more details below), only five singletons were found in the comprehensive assembly, suggesting that the best individual *Csp* assemblies were not missing a significant number of genes.

### Phylogeny to resolve evolutionary relationships between *Csp* samples from woody ornamentals in the United States and related species and genera

**Fig 6:**
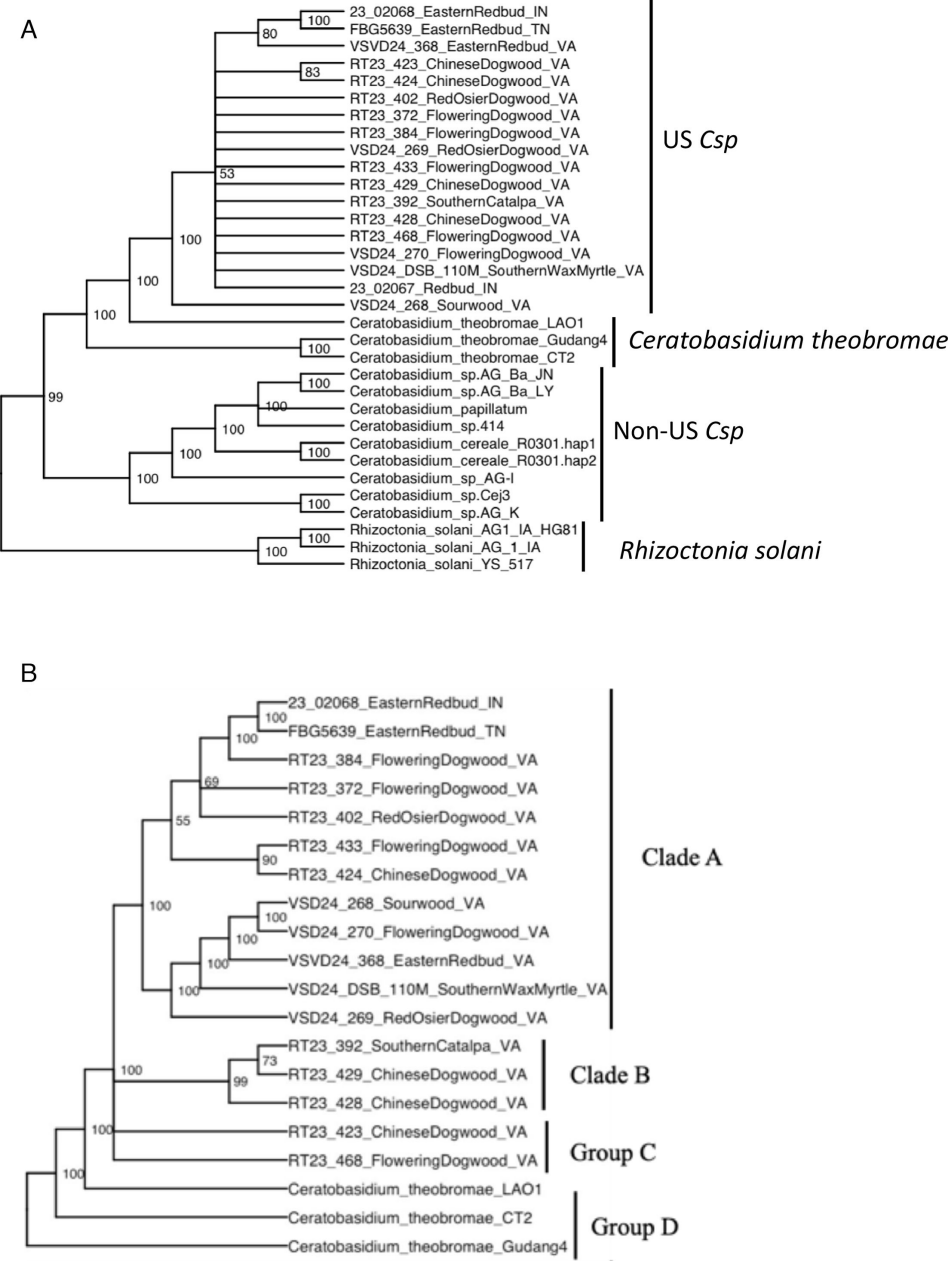
(**A**) Maximum likelihood phylogenetic tree constructed using 76 single-copy orthologous genes from assembled U.S. *Csp* genomes, publicly available *Ceratobasidium* genomes, and *R. solani* isolates used as an outgroup to root the tree (best model: VT + F + I + I + R6). Branch tips are labeled with the sample IDs. Bootstrap support values are shown at each node. (**B**) Core genome phylogenetic tree of *Csp* genomes recovered from woody ornamental hosts in the United States (best model: VT + F + I + I + R6). A total of 1,473 core genes were used to construct the tree, which was rooted on genomes of *Ct* isolates from cacao and cassava in Southeast Asia. Branch tips are labeled with the sample IDs.

To resolve the evolutionary relationships between *Csp* genomes from the United States, other publicly available *Csp* and *Ct* genomes, and related *R. solani* genomes, a core phylogenetic tree was constructed based on 76 conserved single-copy orthologs. Only the 13 U.S. *Csp* genome assemblies with the highest BUSCO scores were used. The resulting maximum likelihood tree (Fig. 6A) revealed a well-supported monophyletic clade composed exclusively of U.S. *Csp* isolates. The relationships among most of these isolates remain unresolved with bootstrap support below 50, leading to a striking polytomy. The Southeast Asian *Ct* genomes (CT2 and Gudang4 from cacao) formed a separate, well-supported clade distinct from the U.S. *Csp* lineage. The distinct branch of the single LAO1 genome from cassava suggests that it may be the only representative of a yet undersampled additional *Ceratobasidium* clade. These genomes all shared a most recent common ancestor distinct from a sister clade of all other *Csp*. Three *R. solani* genomes, chosen to root the tree, formed yet another, well-separated clade. In summary, the core genome tree confirms that the *Csp* isolates from woody species in the United States only recently diverged from a common ancestor and that they are closely related to, but distinct from, the *Ct* isolates from cacao and cassava in Southeast Asia.

### Phylogeny to resolve evolutionary relationships among *Csp* samples from woody ornamentals in the United States

A finer-scale analysis focusing on the 17 U.S. *Csp* assemblies of the highest quality and the three publicly available *Ct* genomes based on 1,473 core genes confirmed the genetic coherence of the U.S. *Csp* population (Fig. 6B). Bootstrap support below 50 at the base of the U.S. *Csp* clade still leads to multifurcation, confirming a recent radiation event from a common ancestor. However, compared to the 76-gene tree in Fig. 6A, the U.S. *Csp* clade in the 1,473-gene tree has more resolution, and a few subclades with high bootstrap support become evident. In most cases, these clades include isolates from different host species such as redbud, dogwood, and wax myrtle, indicating the absence of host adaptation. Since only one isolate from Indiana and one from Tennessee provided enough reads for high-quality assemblies, and all other isolates came from Virginia, nothing can be concluded about regional differentiation.

**Fig 7:**
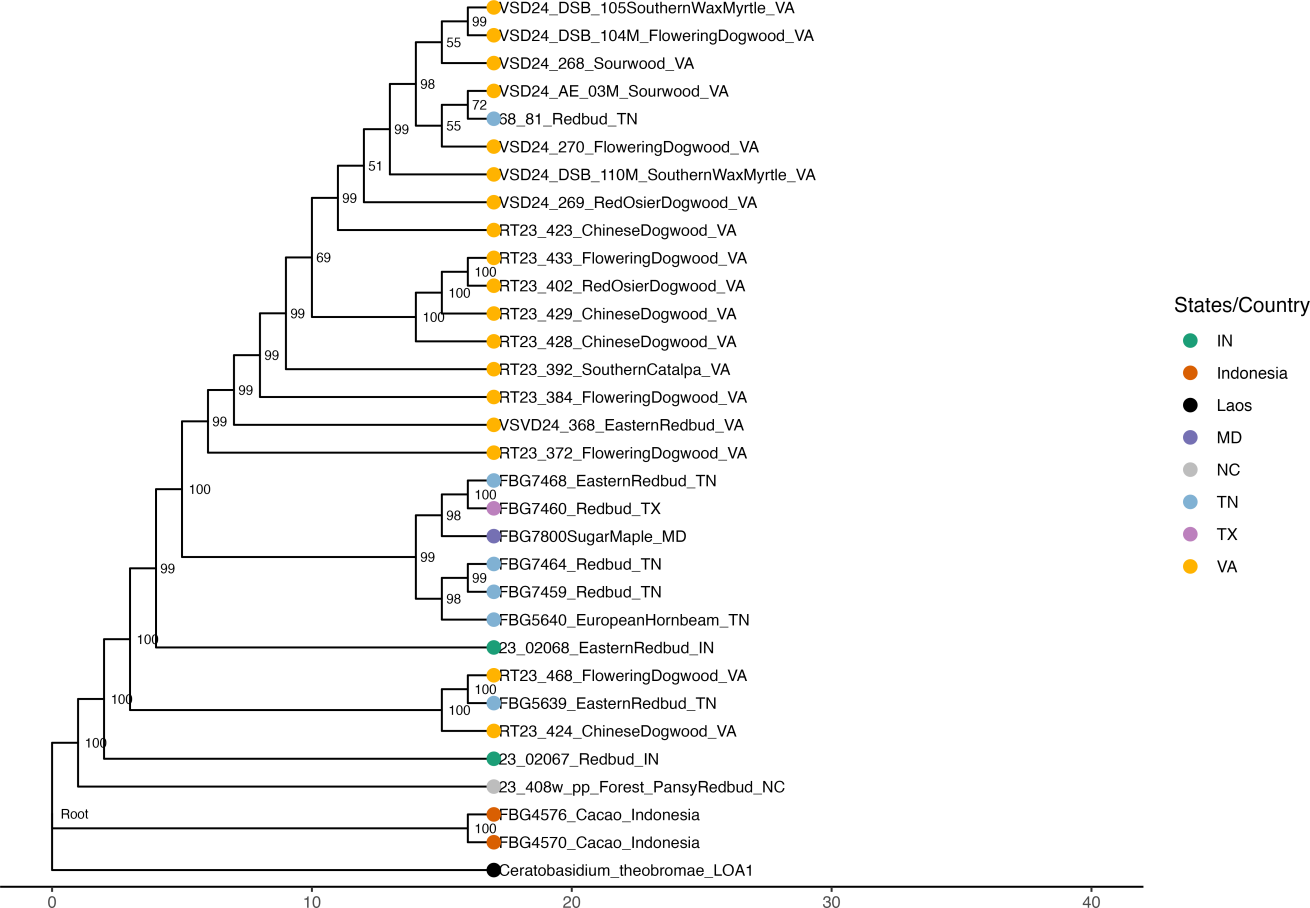
Maximum‐likelihood phylogeny constructed under a GTR + Γ model from 32 *Ceratobasidium* isolates using 1.32 million SNPs identified by the NanoCaller tool by aligning reads against the *Ct* LOA1 genome, which was chosen as the root. Ultrafast bootstrap values (≥50%) are shown at nodes.

Since we only obtained high-quality genome assemblies from 17 out of the 106 total samples, our ability to infer evolutionary relationships of U.S. *Csp* isolates using a core genome approach was limited. Thus, we also tried to use unassembled *Ceratobasidium* reads of a larger number of samples: the 17 samples that had provided high-quality draft assemblies and 14 samples that had provided partial genome assemblies, which included two *Ct* samples from cacao from Indonesia. These reads were aligned against the *Ct* LOA1 genome, the genome of the closest available relative of the U.S. *Csp* lineage based on the above core genome analyses. 1,317,177 SNPs were identified and used for phylogenetic reconstruction (Fig. 7).

The two Indonesian cacao isolates formed a separate clade with 100% bootstrap support, confirming the deep evolutionary split between U.S. *Csp* isolates and Southeast Asian *Ct* isolates seen in the two core genome trees (Fig. 6). All U.S. *Csp* isolates form a single clade, also with 100% bootstrap support and thus also in agreement with the core genome trees. The only North Carolina isolate branched off from the most basal node of this clade, which is in line with the epidemiological finding that trees with VSD symptoms were first observed in North Carolina in 2018, and *Csp* may have spread from there to the other U.S. states. Intriguingly, all but two Virginia isolates form a single well-supported clade, and the majority of Tennessee isolates form a second well-supported clade, suggesting that *Csp* populations in these two states may have started to genetically separate from each other. As already seen in the core genome tree, there are no host-specific lineages, suggesting no adaptation to different host species.

### Exploring gene content differences between genomes of U.S. *Csp* isolates and other *Ceratobasidium* and *Rhizoctonia* isolates, and among U.S. *Csp* isolates

All phylogenetic trees showed that the genomes of the U.S. *Csp* isolates form a single clade that is clearly distinct from the genomes of the Southeast Asian *Ct* isolates from cacao and cassava and all public genomes of other *Ceratobasidium* and *Rhizoctonia* isolates. However, just how different is this U.S. clade compared to all other isolates? To help answer this question, we performed a pangenome analysis. We found a total of 176,561 proteins in 12 *Ceratobasidium* and 3 *R*. *solani* genomes. This data set incorporated the five highest quality individual genome assemblies of our U.S. samples, our comprehensive assembly, the genomes of the three *Ct* isolates from cacao and cassava, three public genomes of *Csp* isolates with the highest BUSCO scores, and the genomes of the three *R. solani* genomes used as outgroup in our core genome phylogeny (Fig. 6A).

**Fig 8:**
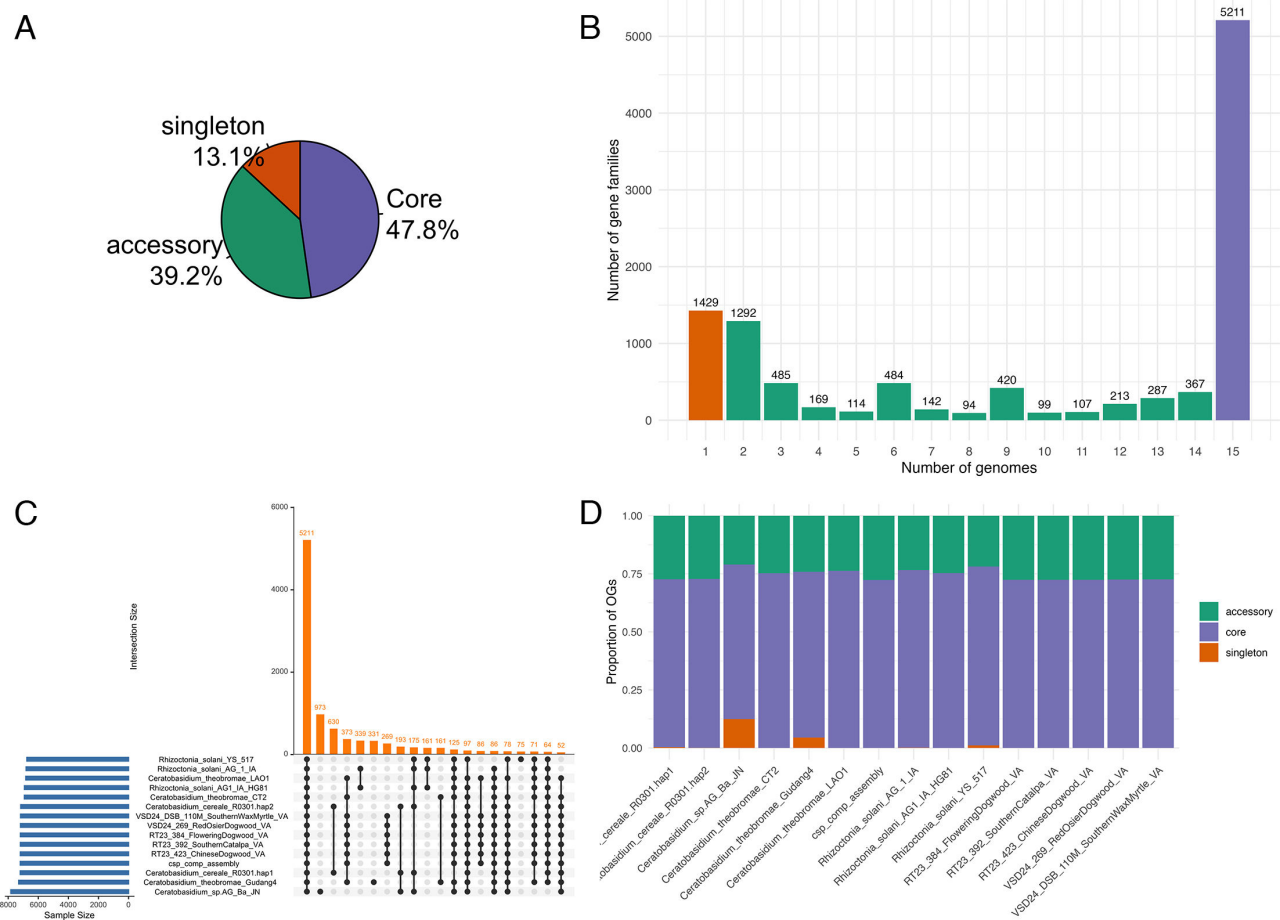
Pangenome analysis. (**A**) Composition of core and accessory orthogroups and singleton genes in the *Ceratobasidium*/*Rhizoctonia* pan-genome. (**B**) Gene accumulation curve (orthogroup count vs. number of genomes): each bar represents how many gene families are present when considering a specific number of genomes. The numbers decrease as more genomes are included, with a sharp increase near the rightmost bars (particularly at 15 genomes), reflecting the core gene families shared by nearly all genomes. This curve illustrates how the pangenome grows and highlights both unique and shared gene content across genomes. (**C**) UpSet plot showing intersections of orthogroups across 15 *Ceratobasidium* and *R. solani* genomes. Bar heights indicate the number of orthogroups for each presence/absence pattern (only intersections with ≥50 OGs are shown), with the connected dots below identifying the genomes included in each intersection. (**D**) Composition of core, accessory, and singleton genes per genome.

The pangenome analysis identified 10,913 non-redundant orthologous protein groups (orthogroups). Of these, 5,211 orthogroups (47.8%) were classified as core, being shared across every single analyzed genome. Additionally, 4,278 orthogroups (39.2%) were categorized as accessory, present in some but not all genomes, reflecting genomic variability within the species. Singletons, representing 13.1% of the orthogroups (1,424), consisted of genes unique to individual genomes, either due to differences in genome quality or representing true differences between individual isolates (Fig. 8A). The gene-accumulation curve (Fig. 8B) plots orthogroup counts against the number of genomes considered. Counts generally decline as *N* increases, with a pronounced spike in the rightmost bin (15 genomes) that reflects the core orthogroups shared by nearly all genomes, while the spread across intermediate bins indicates an open pangenome with substantial accessory variation among isolates.

The UpSet plot (Fig. 8C) shows how the accessory orthogroups are distributed. Importantly, 269 orthogroups were shared among the five genomes derived from US samples and the comprehensive assembly but were absent from all other analyzed genomes, while 161 orthogroups were shared by the two *Ct* genomes from cacao but were absent from all other analyzed genomes. This difference in gene content underscores the evolutionary separation between these two clades. An additional 86 genes were shared among the U.S. *Csp* genomes and the *Ct* LAO1 genome from cassava but were absent from all other genomes, while fewer than 50 genes were shared between the *Ct* LAO1 genome and the two *Ct* genomes from cacao or between the U.S. *Csp* genomes and the two *Ct* genomes from cacao. This further confirms the closer relationship between the U.S. *Csp* isolates with the *Ct* lineage from cassava than with the *Ct* genomes from cacao, which was also evident in the core genome tree in Fig. 6A. Per-genome composition plots (Fig. 8D) further indicate broadly similar proportions of core, accessory, and singleton content across isolates, with modest variation in singleton burden that likely reflects both assembly completeness and genuine lineage-specific gene gain/loss.

To hone in on the genomic diversity among the U.S. *Csp* genomes and their comparison with the *Ct* genomes, we conducted a second pangenome analysis of all 17 *Csp* genomes and 3 *Ct* genomes from the core-genome phylogeny (Fig. 6B). We also included the comprehensive assembly because of the confidence we have in its completeness. Clustering 227,262 proteins from these 20 genomes resulted in 9,902 orthologous groups: 65.1% core, 31.7% accessory, and 3.2% singletons. The UpSet plot (Fig. S2) reveals 6,446 core orthogroups found in all genomes. One can notice that each of the lowest quality genomes, such as *Csp* VSD24-268_Sourwood_VA or 23_02068_EasternRedbud_IN, is missing between 200 and 400 genes, probably because of their low genome completeness. Therefore, the true core genome of the analyzed genomes probably exceeds 7,000 orthogroups. Since we had observed two well-supported clades in Fig. 6B, we were particularly curious to see whether these two clades could be distinguished based on ortholog presence or absence, but no such trend is visible, suggesting that the two clades have only diverged minimally so far.

As for the differences between the U.S. *Csp* genomes and the *Ct* genomes from Southeast Asia, this focused *Csp*/*Ct* pangenome analysis confirmed the substantial differences in orthogroup content found between the two groups in the wider pangenome analysis from Fig. 8, only that the U.S. *Csp* genomes now shared slightly fewer orthogroups, 214 versus 269, probably because of the inclusion of the lower-quality genomes with lower completeness.

### Prediction of effector genes in accessory genomes

For effector prediction, we targeted the accessory orthogroups identified in the 15 genomes used in the phylogenetic analysis (Fig. 6A) and the pangenome analysis (Fig. 8). Accessory proteins per genome ranged from 2,184 in *Ct* LAO1 to 8,485 in *Csp* AG_Ba_Jn (Table 2). Out of the 16,294 accessory proteins, SignalP 6.0 predicted 1,431 to have N-terminal signal peptides. To exclude membrane-bound proteins from this group, we applied TMHMM v2.0c to remove sequences with at least one predicted transmembrane (TM) helix. After this filtering step, 1,231 proteins were retained. One of these proteins was removed because it contained a mitochondrial transit peptide identified by TargetP 2.0. None were found to have ER-retention motifs by ScanProsite (PS00014) or GPI-anchor signals by NetGPI 1.1. Therefore, 1,230 secretome candidates were finally screened with EffectorP 3.0 in fungal mode, which partitioned them into effectors and non‐effectors and further sub‐classified effector candidates as apoplastic, cytoplasmic, or dual‐localized (Table 2).

**TABLE 2:** Summary of accessory gene content and effector predictions for *Ceratobasidium* and *R. solani* genomes

Sample	Accessory genes	Secreted	Effectors	Apoplastic	Cytoplasmic	Dual	Non-effectors
Ceratobasidium_cereale_R0301.hap1	3,067	191	88	50	24	14	103
Ceratobasidium_cereale_R0301.hap2	3,078	195	87	50	25	12	108
Ceratobasidium_sp.AG_Ba_JN	8,485	514	207	105	57	45	307
Ceratobasidium_theobromae_CT2	2,241	171	79	32	20	27	92
Ceratobasidium_theobromae_Gudang4	4,073	322	149	64	48	37	173
Ceratobasidium_theobromae_LAO1	2,184	178	82	39	26	17	96
csp_comp_assembly	3,281	691	312	144	83	85	379
Rhizoctonia_solani_AG_1_IA	2,211	161	73	28	23	22	88
Rhizoctonia_solani_AG1_IA_HG81	2,355	170	74	33	23	18	96
Rhizoctonia_solani_YS_517	2,607	191	93	45	25	23	98
RT23_384_FloweringDogwood_VA	2,787	200	94	45	26	23	106
RT23_392_SouthernCatalpa_VA	2,723	203	94	46	28	20	109
RT23_423_ChineseDogwood_VA	2,712	198	95	40	31	24	103
VSD24_269_RedOsierDogwood_VA	2,690	203	90	41	21	28	113
VSD24_DSB_110M_SouthernWaxMyrtle_VA	2,691	219	98	53	21	24	121

To identify clade-specific effectors, we analyzed the predicted effector repertoires across the major clades identified in our core-genome phylogeny (Fig. 6A). The non-U.S. *Csp* clade contained the highest number of unique predicted effectors, with 150 effectors specific to this group. The U.S. *Csp* clade harbored a distinct set of 34 unique effectors, while the *Ct* (cacao/cassava) clade had 52 unique effectors. The *R. solani* group displayed the lowest effector count, with only 10 identified effectors. These results reveal substantial differences in effector content between the U.S. *Csp* clade and its sister clades, yielding a distinct set of effector candidates that can be further investigated for their potential roles in host specificity and virulence.

## DISCUSSION

Since the first detection of *Csp* on an eastern redbud with VSD symptoms in Tennessee in 2019, VSD has quickly spread across 13 U.S. states and *Csp* has been detected molecularly on 50 genera of woody ornamentals (7, 65). However, the fastidious nature of *Csp* has prevented Koch’s postulates from being completed and from establishing *Csp* as the sole causative agent of VSD (6, 15, 66). Therefore, the first objective of this study was to determine whether any other bacterial, fungal, or oomycete pathogen besides *Csp* is consistently associated with VSD and may contribute to the disease.

Metagenomic sequencing is a promising approach to answer this question since it does not require culturing and can potentially identify all microbes in a sample to the species rank, and, if enough sequencing data are obtained, MAGs can be obtained and used in phylogenetic reconstruction (67, 68). However, while metagenomics has become relatively established for the identification of bacterial pathogens, including plant pathogens (69–71), identification of fungi from metagenomic data is challenging and is still in its infancy (72–74). One main issue is that taxonomic profiling of metagenomic sequences heavily relies on the availability of correctly classified reference genomes, and only 659 fungal genomes are currently (July 2025) included in NCBI’s manually curated RefSeq database compared to 435,623 prokaryotic genomes (75). Therefore, instead of using the standard RefSeq-based reference database provided by the developers of the taxonomic profiler Kraken2, we built a custom fungal and oomycete Kraken2 database using all genomes from NCBI’s Assembly database. However, since curation by NCBI of the Assembly database is limited, genomes may be misclassified, leading to errors in the Kraken2 database constructed from these genomes and, ultimately, giving false-negative and false-positive results. This is probably the reason that several fungal species identified in our metagenomes by Kraken2 were not confirmed when using other tools (sourmash and minimap2). The same problem of false positives even happened when using Kraken2 with a standard RefSeq-based database for identification of bacterial species, possibly a result of misclassified genomes even present in NCBI’s RefSeq database (76, 77) and/or limitations inherent to the Kraken2 algorithm (28–30). Therefore, improved Kraken2 reference databases are urgently needed, and it is key not to rely on a single taxonomic profiler when analyzing metagenomic data.

Importantly, though, we were able to identify sequencing reads as *Csp* in all samples using both Kraken2 in combination with our custom fungal database (which included *Ct* genomes while the standard RefSeq-based Kraken2 database did not) and mapping to two *Ct* reference genomes using minimap2. We obtained a similar number of reads with both tools, giving us confidence that the number of false-positive and false-negative reads assigned to *Csp* by these tools was limited. However, independently of the bioinformatics tools, we cannot exclude that a small number of *Csp* sequences were the result of barcode hopping, whereby some DNA molecules from one sample get erroneously labeled with the barcode from another sample, as has been reported to occur at low frequency with nanopore sequencing (78).

Since no pathogen besides *Csp* was consistently detected in the plant samples, our results confirmed that no other pathogen beyond *Csp* plays a significant role in VSD. The presence of the canker and dieback pathogen *B. dothidea* in some samples suggests that this pathogen may occasionally contribute to the severity of VSD, but symptoms caused by *B. dothidea* were previously found to be distinct from VSD, and the pathogen was excluded from being the VSD-causing agent (65). The bacterial species *Stenotrophomonas maltophilia*, sometimes reported as a plant pathogen (79), was mainly identified in mycelium samples but not in plant samples and can thus also be excluded as causing VSD.

An unexpected result was the low number of *Csp* reads in many of the plant samples. We speculate that in some cases, samples may have been taken from tissue with low pathogen abundance or late during infection (with *Csp* DNA already degraded by the time of sampling), or that the employed DNA extraction protocols were relatively inefficient in releasing DNA from *Csp* cells compared to releasing DNA from other fungal or bacterial cells. Moreover, metabolites present in woody tissue are known to interfere with DNA extraction (80). Thus, for sensitive detection of *Csp*, it is important to still improve DNA extraction protocols. Finally, it is important to point out that taxa for which closely related genomes have not been published (and are thus missing from our databases) may have been present but remained undetectable.

After we had confirmed *Csp* as the VSD pathogen with the metagenomic results, we attempted assembly of *Csp* genomes from as many samples as possible for downstream core genome phylogenetic and pangenome analyses. For including genomes in downstream analysis, we used a minimum completeness score threshold of 70%, based on the Agaricomycetes BUSCO data set (81). This may seem a relatively low completeness score, but even our comprehensive assembly with a genome coverage of 250× (i.e., every region of the assembly was covered on average by 250 reads, making it very unlikely that some genomic regions were not covered at all) only reached a completeness score of 83.7. This suggests that the *Ceratobasidium* genus may be missing some of the genes present in most of the other sequenced Agaricomycetes genomes or that some genomic regions either escaped nanopore sequencing or could not be assembled. However, since our average read length was just shy of 3,000 bp, and nanopore sequencing is particularly well-suited for assembling structurally complex regions and repeat regions (20, 82, 83), this seems unlikely. Also, since the pangenome analysis only identified one singleton gene in the comprehensive assembly and only between 0 and 38 singletons in the 12 best individual assemblies (which had completeness scores of 83%–84% based on BUSCO agaricomycetes_odb10), we conclude that our best individual assemblies have higher completeness than what the BUSCO scores suggest. Moreover, the pangenome results suggest that the genome assemblies present very low levels of contamination with other microbial sequences or plant sequences, and that sequencing errors did not significantly affect gene calling. Finally, the highly similar gene content between the best individual *Csp* assemblies (assembled from reads that mapped to genomes of the related, yet distinct, Southeast Asian *Ct* lineage) and the comprehensive assembly (which was based on all non-plant reads from five samples) shows that when using long reads, assembling genomes from reads mapped to a related genome has a low risk of accidentally excluding genomic regions that are possibly absent from the related genome used for mapping.

Once the genome assemblies were obtained, we built one 76-gene core genome tree to investigate the evolutionary relationships between the *Csp* genomes from the United States and related *Ceratobasidium* and *Rhizoctonia* species from other world regions, and one 1,473-gene core genome tree to unravel the evolutionary relationships among the *Csp* genomes from the United States. The results from the first tree mainly confirmed a previously published tree based on the ITS, LSU, ATP6, TEF1, and RPB2 genes (6). However, probably because of the higher number of loci used (76 compared to 5), a single node at the base of all *Ceratobasidium* genomes had high bootstrap support, confirming that the *Ceratobasidium* genus is monophyletic, while the 5-gene tree could not separate the *Ceratobasidium* genomes from the *Rhizoctonia* genomes. Moreover, the 76-gene tree subdivided the *Ceratobasidium* clade into two strongly supported subclades: one consisting of the Southeast Asian *Ct* genomes, and the U.S. *Csp* genomes and the other consisting of all other *Csp*. Interestingly, both the 76-gene tree and the 1,473-gene tree provide strong support for the *Csp* genomes from the United States sharing a more recent common ancestor with the *Ct* LAO1 genome from cassava in Laos than with the *Ct* genomes from cacao in Indonesia. Because of the small number of genomes that were available, this may be a coincidence. However, the result aligns with the earlier finding that the ITS regions of *Csp* isolates in the United States are identical to ITS sequences of *Ceratobasidium* isolates from Japanese honeysuckle in China (6), of which Laos is a direct neighbor, further suggesting that the U.S. *Csp* lineage may have its origin in that geographic region.

Regarding the evolutionary relationships among U.S. isolates, the 76-gene tree did not provide support for any subclades. This is not surprising since their most recent common ancestor may have existed not much longer than 7 years ago, when the first trees with VSD symptoms were observed. Therefore, a few mutations may have accumulated in the 76 core genes since then. The 1,473-gene tree did reveal two well-supported individual clades, both including samples from several different plant genera, suggesting no adaptation to different hosts. Because only two clades had strong bootstrap support, and since most of our best assemblies were of samples collected in one geographic region of Virginia, we could not make any conclusions about possible dissemination pathways. Therefore, we attempted construction of an SNP-based phylogeny based on alignment of unassembled individual reads against the *Ct* LAO1 genome. Since assembly was not required, this allowed us to include many more samples. While ONT significantly reduced its sequencing error rate over the years, it is still around 1% (84, 85). Probably because of this relatively high error rate, even when using the most stringent SNP-calling parameters, an excessively high number of 1.32 million SNPs were called. Random sequencing errors are expected to occur independently in each genome and are not expected to falsely group genomes together into clades. However, this high number of SNPs still reduced our confidence in the obtained SNP tree (even though bootstrap support for most clades was very high). Therefore, although the location of the only North Carolina sample at the very base of the U.S. clade (suggesting that it is most similar to the most recent common ancestor of all U.S. isolates) agrees with the finding that VSD was first observed in North Carolina and *Csp* may have spread to the rest of the U.S. from there, this result will need to be followed up on. Additional sampling and sequencing, ideally using technologies with lower error rates, such as Illumina or PacBio HiFi, should be performed. Similarly, the possible separation of *Csp* into a Virginia and a Tennessee sub-population also still needs to be confirmed.

The final question we wanted to answer was: are there any signs in the genomes of the U.S. *Csp* isolates that suggest that the *Csp* lineage adapted to woody ornamentals and the North American climate and, possibly, represents a new *Csp* distinct from *Ct*? Our pangenome and effector gene prediction analyses found substantial differences in gene content between the genomes in the U.S. *Csp* clade and genomes in its sister clades. The U.S. *Csp* clade contained 214–269 unique genes, depending on which genomes were compared. Of these, up to 34 genes were predicted to encode secreted *Csp*-specific effectors, which possibly contribute to virulence by suppressing plant immunity or degrading plant cell walls, typical mechanisms used by fungal plant pathogens (86, 87). Unfortunately, following up on these findings with functional studies of pathogenicity and host specificity (16, 88) will be complicated because of the fastidious nature of *Csp*, making it unlikely to unravel the basis of the exceptionally wide host range of the U.S. *Csp* lineage any time soon.

Interestingly, the *Ct* lineage presented by the only available LAO1 genome from cassava in Laos is not only more closely related to the U.S. *Csp* lineage based on phylogeny but also shares more genes with the U.S. *Csp* lineage than the cacao lineage, further suggesting that the U.S. *Csp* lineage may have originated from a pathogen population located in geographic proximity to Laos. Therefore, the question about speciation is more complex than we anticipated. We may either have just one *Ct* species, including *Ct* from cacao and from cassava, and *Csp* from ornamentals in the United States, two species (either grouping *Ct* from cassava in Laos with the U.S. *Csp* lineage or with the *Ct* lineage from cacao), or three separate species. Only a more in-depth taxonomic analysis, including additional genome-sequenced isolates and possibly phenotypic analyses, will be able to answer this question.

In conclusion, this study revealed how metagenomics using long-read sequencing can overcome the challenges involved with the absence of pure cultures when dealing with fastidious fungal pathogens. While we encountered many issues with false positives during taxonomic profiling, the use of multiple, parallel approaches and a custom database still allowed us to confidently conclude that *Csp* is the only causative agent of VSD in U.S. ornamentals. Also, the long reads allowed for assembly of several high-quality draft genomes, giving insight into the relationships between the U.S. *Csp* lineage and related pathogens. On the other hand, the still relatively high error rate of ONT reduced our confidence in the obtained SNP-based tree, which has the potential to give additional insights into pathogen dissemination patterns. Also, our high-quality genomes allowed us to perform a solid pangenome analysis, providing the basis for a future taxonomic investigation, functional studies of pathogenicity and host range, and development of new molecular markers for highly specific, high-throughput detection assays.

## Data Availability

The metagenomic sequencing data generated in this study have been deposited in the NCBI Sequence Read Archive (SRA) under BioProject accession number PRJNA1290818. A total of 106 sequence data sets is available, with associated BioSample metadata provided in the SRA submission. Custom scripts, pipelines, intermediate files, and databases used for analysis are publicly available in permanent Zenodo repositories: (i) core scripts, intermediate tables, and figure notebooks: https://doi.org/10.5281/zenodo.17344926, (ii) single-nucleotide polymorphism (SNP) call files: https://doi.org/10.5281/zenodo.17315410, (iii) Kraken2 classification outputs: https://doi.org/10.5281/zenodo.17220370, and (iv) Hash (sourmash) database for fungi and oomycetes database: https://zenodo.org/records/17527097. Note that for the Kraken2 database, we only provided a combined_mapping.txt file in the scripts directory with a list of genomes instead of the formatted database since the file size exceeded Zenodo’s file size limit.

## SUPPLEMENTAL MATERIAL

Supplemental figures spectrum.02523-25-s0001.pdf Figures S1 and S2.

Table S1 spectrum.02523-25-s0002.xlsx Metadata and metagenomic sequencing summary for all samples analyzed in this study, including host plant identity, geographic location, disease symptoms, sequencing statistics, and read-based abundance of the dominant fungal, oomycete, and bacterial taxa.

Table S2 spectrum.02523-25-s0003.xlsx Kraken2- and sourmash-based taxonomic assignment, read-based abundances, and BLAST identification for metagenomic sequences assigned to major fungal taxa across samples.

Table S3 spectrum.02523-25-s0004.xlsx Kraken2- and sourmash-based taxonomic assignment, read-based abundance, and BLAST identification for metagenomic sequences assigned to major bacterial taxa across samples.

Table S4 spectrum.02523-25-s0005.xlsx Genome assembly statistics for *Ceratobasidium* isolates, including read depth, mapping metrics, contig and scaffold characteristics, GC content, coverage, and BUSCO-based completeness assessments for each assembly.

Table S5 spectrum.02523-25-s0006.xlsx BLAST-based taxonomic assignments and coverage statistics for all *Ceratobasidium*-associated contigs and the subset of selected contigs, including accession hits, alignment metrics, organism names, and depth estimates across multiple mapping tools.

ASM does not own the copyrights to Supplemental Material that may be linked to, or accessed through, an article. The authors have granted ASM a non-exclusive, world-wide license to publish the Supplemental Material files. Please contact the corresponding author directly for reuse.
